# The roles of connectivity and neuronal phenotype in determining the pattern of α-synuclein pathology in Parkinson's disease

**DOI:** 10.1016/j.nbd.2022.105687

**Published:** 2022-03-10

**Authors:** Michael X. Henderson, Martin T. Henrich, Fanni F. Geibl, Wolfgang H. Oertel, Patrik Brundin, D. James Surmeier

**Affiliations:** a Parkinson's Disease Center, Department of Neurodegenerative Science, Van Andel Institute, Grand Rapids, MI 49503, United States of America; b Department of Neurology, Philipps-University Marburg, Marburg 35043, Germany; c Department of Psychiatry and Psychotherapy, Philipps-University Marburg, Marburg 35043, Germany; d Department of Neuroscience, Feinberg School of Medicine, Northwestern University, Chicago, IL 60611, United States of America

**Keywords:** Vulnerability, Neurodegeneration, Lewy pathology, Synaptic, Propagation, SNCA

## Abstract

Parkinson's disease (PD) is the most common neurodegenerative movement disorder, and motor dysfunction has been attributed to loss of dopaminergic neurons. However, motor dysfunction is only one of many symptoms experienced by patients. A neuropathological hallmark of PD is intraneuronal protein aggregates called Lewy pathology (LP). Neuropathological staging studies have shown that dopaminergic neurons are only one of the many cell types prone to manifest LP. Progressive appearance of LP in multiple brain regions, as well as peripheral nerves, has led to the popular hypothesis that LP and misfolded forms of one of its major components – α-synuclein (aSYN) – can spread through synaptically connected circuits. However, not all brain regions or neurons within connected circuits develop LP, suggesting that cell autonomous factors modulate the development of pathology. Here, we review studies about how LP develops and progressively engages additional brain regions. We focus on how connectivity constrains progression and discuss cell autonomous factors that drive pathology development. We propose a mixed model of cell autonomous factors and trans-synaptic spread as mediators of pathology progression and put forward this model as a framework for future experiments exploring PD pathophysiology.

## 1. Introduction

Parkinson's disease (PD) is characterized by the gradual appearance of α-synuclein (aSYN)-laden intraneuronal inclusions, referred to as Lewy pathology (LP) (Goedert et al., 2013), and neurodegeneration in certain brain regions. For the last two decades, one of the dominant theories about the origin of this pathology is that LP spreads through synaptic coupled networks from the periphery, inducing neurodegeneration and the symptoms of PD (Brundin and Melki, 2017). Braak and colleagues initially advanced this hypothesis based upon post-mortem examination of the brains of premanifest and symptomatic PD patients using immunocytochemical localization of aSYN aggregates (Braak et al., 2003). Since then, there has been an explosion of experimental studies supporting the proposition that aSYN pathology can spread from one neuron to another in the brain. Much of this work has been driven by the discovery by Lee and colleagues that the pathology induced by injection of fibrillar forms of aSYN into the rodent brain readily spreads from the site of inoculation (Luk et al., 2012). Subsequent work by Recasens et al. found a similar phenomenon in the primate brain following inoculation with material derived from human LP, solidifying the connection to PD (Recasens et al., 2014). Because spreading of aSYN pathology depends upon the recruitment of endogenous aSYN to misfolded aSYN templates, the term ‘prion-like’ has commonly been used to describe this phenomenon (Brundin and Melki, 2017).

Although attractive in its simplicity, there are many unresolved questions about how misfolded forms of aSYN spread and the relevance of experimental studies to the human condition. The goal of this review is to rigorously explore some of these questions, critically discuss the existing literature and to identify key gaps in our understanding. There are four sections to the review. First, we discuss the evidence from clinical autopsy studies suggesting that aSYN pathology is triggered in restricted areas of the nervous system and then spreads along the synaptic connectome. Second, we discuss observations in model systems consistent with the proposition that aSYN pathology can propagate transsynaptically in a prion-like fashion, and the relationship between these studies and human autopsy data. Third, we discuss potential mechanisms mediating seeding and propagation of aSYN pathology in the brain, as well as neurodegeneration, highlighting the potential role of cell autonomous factors. Last, we consider the possibility that the LP characteristic of PD reflects a combination of trans-synaptic spread of aSYN aggregates and cell autonomous factors.

## 2. Evidence for trans-synaptic propagation of aSYN pathology in humans

LP was first described in 1912 by Friedrich H. Lewy by classical histological stains (Lewy, 1912). Later, ultrastructural studies and immunohistochemistry showed that LP consisted of aSYN fibrils and various organelles, including mitochondria, membranes and cellular debris (Duffy and Tennyson, 1965; Forno, 1996; Shahmoradian et al., 2019; Spillantini et al., 1998b; Spillantini et al., 1997). These studies have shown that fibrillar aSYN is a significant protein component of LP and possible seed for PD progression. Several lines of evidence now suggest that LP in PD is seeded either in the periphery or in the brain and then spreads. One line of evidence comes from the progression in symptoms associated with PD. For example, James Parkinson noted that constipation was an early and consistent complaint of people affected by “paralysis agitans”, implicating gastrointestinal or brainstem pathology in prodromal PD (Parkinson, 2002). More recently, longitudinal studies of PD patients suggest that deficits in olfactory sensation and gastrointestinal function may appear decades before a clinical diagnosis of PD (which requires manifest motor disability) (Fereshtehnejad et al., 2019; Postuma and Berg, 2019; Schaeffer et al., 2020). Although these symptoms have a relatively low specificity for PD, it is suggestive of a prolonged prodromal period in which LP is accumulating. Even within the basal ganglia structures, whose dysfunction underlies the cardinal motor symptoms of PD, pathology may be accumulating well before motor symptoms. At the time of diagnosis, there already has been a dramatic loss of proteins associated with dopaminergic transmission in the striatum and significant loss of phenotypic markers in the substantia nigra itself (Cheng et al., 2010; Fearnley and Lees, 1991; Greffard et al., 2006; Kordower et al., 2013). It is highly likely that these changes started decades before and that the basal ganglia circuitry has compensated for declining dopaminergic dysfunction until a ‘tipping point’ is reached (Bezard et al., 1999; Bezard et al., 2001; Nandhagopal et al., 2011). Even after diagnosis, there is often a progressive decline in cognitive function, and a high percentage of PD patients progress to dementia (Aarsland et al., 2003).

Another line of evidence that is consistent with the spreading hypothesis comes from autopsy data. Identification of aSYN as a major protein component of LP (Spillantini et al., 1998a; Spillantini et al., 1997) led to a detailed immunocytochemical analysis of its distribution in post-mortem brains. Based upon this kind of analysis of ‘pre-symptomatic’ and PD brains at varying times after diagnosis, Braak and colleagues (Braak et al., 2003) advanced the hypothesis that in the prodromal stages of PD, LP is found in the dorsal motor nucleus of the vagal nerve (DMV), locus coeruleus (LC) and olfactory system structures. At the time of diagnosis (stage 3), the distribution of LP expands in the brainstem, the substantia nigra pars compacta (SNpc), the diencephalon and the basal forebrain. In later stages (4–6), LP worsens in those locations where it is seen previously and expands to include regions of the telencephalon. This broadening of LP and neurodegeneration is correlated with worsening of motor symptoms, and cognitive decline (Braak et al., 2006b; Irwin et al., 2017; Irwin et al., 2012).

Braak and others followed up this initial staging with investigations of the peripheral nervous system and found that LP can be found in the gastrointestinal tract in regions innervated by the DMV through the vagal nerve (Braak et al., 2006a). This finding is consistent with a peripheral seeding of LP that spreads to the brain. But it is important to note that only about half of PD cases follow the Braak staging (Beach et al., 2009). The alternative patterning of LP is consistent with the seeding of LP in the brain itself, which then spreads (Borghammer, 2021; Schaeffer et al., 2020; Borghammer, 2021).

Recently, imaging studies have provided tentative support for Braak staging using brain atrophy as a proxy (Dagher and Zeighami, 2018; Laansma et al., 2021; Pandya et al., 2019). In both cross-sectional and longitudinal studies, brain atrophy in PD patients is progressive and correlated with connectivity (Dagher and Zeighami, 2018; Pandya et al., 2019). Modeling these data have allowed inferences to be drawn about potential sites of disease origin (Dagher and Zeighami, 2018), and may allow a personalized approach to disease progression (Brown et al., 2019). Specifically, if the brain region of disease origin is known and the parameters controlling disease progression are well-defined, then physicians could provide a more personalized disease prognosis for patients and possibly identify populations who would be most likely to benefit from therapies targeted to specific brain regions or cell types. While the relationship between regional atrophy and underlying disease mechanisms remains to be determined, having an imaging ligand for LBs would at least allow an assessment of its potential pathogenic role.

Another set of observations that are consistent with the transmissibility of LP come from post-mortem studies of the brains of patients who previously received grafts of fetal midbrain dopamine neurons. These revealed the presence of LP-like aggregates in young dopaminergic neurons inside the transplants (Kordower et al., 2008a; Li et al., 2008). Specifically, several cases have now been documented showing that 2–12% of grafted dopaminergic neurons exhibit aSYN aggregates between 10 and 24 years after transplant surgery (Brundin and Kordower, 2012; Kordower et al., 2008a; Kordower et al., 2008b; Kurowska et al., 2011; Li et al., 2008; Li et al., 2016). Notably, such aggregates were not observed in patients who died 2–5 years after surgery (Kordower et al., 1995; Kordower et al., 1996; Mendez et al., 2005; Olanow et al., 2003), which suggests that a lag-period was required for the LP to develop in the young, transplanted neurons. The cytosolic levels of aSYN, presumably in a soluble form, were also increased in 40% and 80% of the grafted neurons at 12- and 16-years post-grafting, respectively (Li et al., 2008). These increased levels of cytosolic aSYN resemble those seen in normal aging brains (Chu and Kordower, 2007) and suggest that the grafted neurons might be more susceptible to seeding of aggregates by internalization of host-brain derived aSYN assemblies. However, there are caveats to this interpretation. One is that, as described below, dopaminergic neurons create a cellular environment that is conducive to the formation of aSYN aggregates, particularly in stressful situations, as those that might be found in a graft.

Despite the evidence that LP spreads in the brain through the course of PD, it appears that some regions are more vulnerable to disease than others. Thus, the pattern of LP is restricted in distribution and does not appear in many regions that are synaptically coupled to those that manifest LP early in the disease (Surmeier et al., 2017). Within nuclei manifesting LP, typically only a small percentage (5–15%) of neurons manifest LP even at end stage (Braak and Del Tredici, 2009; Dugger and Dickson, 2010). It is unclear whether this is due to LP formation being stochastic, to these cells being particularly vulnerable to development of LP, or to whether other cells that developed LP earlier have already died and are therefore not available for analysis (Greffard et al., 2010). The uneven distribution of LP between brain regions and between neurons within a given anatomical structure suggests that there might exist cell- and region-specific factors that modulate susceptibility to degeneration or that individual neurons might be more resilient to LP than their immediate neighbors.

## 3. Evidence for propagation of aSYN pathology in model systems

While it is currently not possible to longitudinally track the evolution of aSYN pathology in PD patients, cell and animal models have shown that misfolded conformers of aSYN can be released and internalized by neurons, inducing the misfolding of endogenous aSYN. This process is commonly referred to “prion-like” because unfolded endogenous proteins are induced to form a misfolded aggregate by an exogenous template.

In a test tube, concentrated aSYN monomer can form elongated fibrils (Conway et al., 1998; Giasson et al., 1999). Following this discovery, it was shown that in cell culture aSYN pre-formed fibrils (PFFs) can be internalized and template misfolding of endogenous aSYN into inclusions (Luk et al., 2009; Volpicelli-Daley et al., 2011). These inclusions are LP-like in that they accumulate detergent-insoluble phosphorylated aSYN, ubiquitin, p62, and other LP-associated proteins (Henderson et al., 2017; Mahul-Mellier et al., 2020; Rey et al., 2016b; Volpicelli-Daley et al., 2011). More recent ultrastructural analyses have shown that PFF-induced inclusions in primary neurons are similar in many ways to LP in human brain (Lashuel, 2020; Mahul-Mellier et al., 2020; Shahmoradian et al., 2019) and can cause neuronal death (Luna et al., 2018; Volpicelli-Daley et al., 2011). The aSYN pathology in neurons induced by PFFs also can spread from cell-to-cell as demonstrated by studies in microfluidic chambers (Tran et al., 2014).

Induction of LP-like aggregates in neuron cultures has enabled the assessment of several cell autonomous mediators of aSYN pathology. For example, neurons lacking endogenous aSYN will not develop inclusions following treatment with PFFs (Henderson et al., 2017; Volpicelli-Daley et al., 2011). Therefore, aSYN expression level is a cell autonomous factor mediating LP vulnerability. Several other cell autonomous factors modulate aSYN pathology. For example, cell surface proteins can bind misfolded aSYN and promote internalization (Aulic et al., 2017; Emmenegger et al., 2021; Mao et al., 2016). Reduced lysosomal integrity due to glucocerebrosidase inhibition or pH disruption can elevate susceptibility to PFFs (Henderson et al., 2020; Karpowicz Jr. et al., 2017). Protein kinases involved in axonal trafficking also may influence how well a cell handles toxic forms of aSYN (Henderson et al., 2017). Neuronal spiking enhances the release of aSYN and the internalization of misfolded aSYN (Ueda et al., 2021; Wu et al., 2020), potentially increasing vulnerability.

Animal models also have provided important insights into the mechanisms underlying LP formation and spreading. PFFs can be stereotaxically injected into the brains of rodents or primates, inducing LP-like aggregates (Luk et al., 2012; Masuda-Suzukake et al., 2013). The most studied model is one in which PFFs are injected into the striatum where the highly arborized axons of vulnerable dopaminergic neurons terminate. In this model, aggregates of detergent-insoluble, phosphorylated aSYN and other LP components (Luk et al., 2012; Masuda-Suzukake et al., 2013) form earliest in the SNpc dopaminergic neurons and other regions of the brain that project to the striatum (e.g., cerebral cortex). In SNpc dopaminergic neurons, formation of aggregates is coupled to cell death. The ability to induce LP-like pathology in a wide range of animal models has been a major advantage of the PFF model. For example, PFF injection in wild-type animals has demonstrated that misfolded forms of aSYN can propagate in the normal brain, establishing relevance of the phenomenon to PD.

The spatiotemporal control of aSYN pathology afforded by localized stereotaxic injection of PFFs into animal brains has enabled the generation of detailed regional maps of the resulting LP-like pathology and how they change as a function of time following the initial seeding event. Several recent studies have combined brain-wide pathology assessment in PFF-treated mice to assess the determinants of spreading (Henderson et al., 2019b; Henrich et al., 2020; Mezias et al., 2019). For example, one study employing intrastriatal PFF injection used a model based on linear diffusion to show that brain-wide pathology followed largely retrograde neuroanatomical connections (Henderson et al., 2019b). Differences between estimated spread through connectivity and regional pathology were partially accounted for by variation in the expression level of aSYN.

To determine if the seeding site affected spreading, another recent study injected PFFs into the pedunculopontine nucleus (PPN), a region that exhibits LP early in the course of PD (Henrich et al., 2020). The authors found that S129 aSYN immunoreactivity following small PFF injections was almost exclusively in cholinergic neurons and difficult to detect in neighboring glutamatergic and GABAergic projection neurons, as is the case in human PD. Using monosynaptic rabies virus mapping approaches to quantitatively map inputs specifically to cholinergic projection neurons, they found that all neurons innervating these ‘starter’ neurons manifested aSYN pathology (pS129 aSYN immunoreactivity) within 6 weeks of the injection. However, in many of these regions, aSYN pathology peaked and then declined or completely cleared within 12 wks, suggesting pathology was transient. Importantly, there was no significant correlation with the nominal strength of the projection and semi-quantitative scoring of PFF-induced pathology. That said, the monosynaptic rabies virus approach does not provide a measure of the functional strength of synapses or their activity, both of which might be factors in propagation (Ueda et al., 2021).

Other studies also have suggested that aSYN pathology might decline following longer survival times (typically many months) after the initial PFF injection, but it is not yet clear whether this is due to the LP-like pathology being cleared by the neurons or if the affected neurons die, leading to fewer aSYN aggregates being visible at the time of sacrifice (Rey et al., 2017). Mice with defective mitochondrial function, due to the absence of one allele of the Engrailed1 transcription factor, also display exacerbated aSYN pathology following intrastriatal PFF injection (Chatterjee et al., 2019) (see below). Another important, but relatively unexplored, determinant of pathological aSYN spreading is inflammation. With stereotaxic injection of foreign material into the brain there is invariably damage and inflammation, which may contribute to spreading and neurodegeneration (Duffy et al., 2018; Rey et al., 2016a; Zheng and Zhang, 2021). A closely related question is the dose-response relationship of aSYN pathology and spreading. One interesting study that has addressed this question found that this relationship was non-linear, and dependent upon the brain region as well as the species of the host (Abdelmotilib et al., 2017). Finally, the conformation of aSYN fibrils should be considered. Several studies have found remarkable differences in the pathogenicity of aSYN derived from multiple system atrophy and PD brains (Peng et al., 2018; Prusiner et al., 2015; Recasens et al., 2014; Tanudjojo et al., 2021). Furthermore, experiments using different strains of recombinant aSYN can seed aSYN pathology differentially in mouse brain (Rey et al., 2019). While recombinant aSYN PFFs induce Lewy-like pathology in mice, differences between mouse models and human disease may be attributable to differences in fibril conformation (Schweighauser et al., 2020).

## 4. Evidence for a cell autonomous contribution to aSYN pathology

As noted above, the distribution of LP in PD, particularly in its early and middle stages is not random but is restricted to a set of nuclei. Notably, nuclei adjacent to those that manifest LP are typically not affected in PD. For example, the largest synaptic input to LC – a nucleus with early and robust LP – comes from the cerebellum; but the cerebellum is devoid of LP (Braak et al., 2003; Schwarz et al., 2015). The striatum, globus pallidus and subthalamic nucleus robustly innervate vulnerable SNpc dopaminergic neurons but manifest few Lewy bodies themselves (Beach et al., 2009; Braak et al., 2003; Halliday et al., 2011). LP in the SNpc does not spread to the substantia nigra pars reticulata (SNr), a few hundred microns away and even though dopamine-releasing dendrites of the most vulnerable ventral tier dopaminergic neurons heavily innervate the SNr itself (Beach et al., 2009; Braak et al., 2003). Even within nuclei that manifest LP, there is heterogeneity. For example, in the PPN, LP is almost exclusively in cholinergic neurons and does not appear in interdigitated GABAergic and glutamatergic neurons (Giguere et al., 2018; Pienaar et al., 2013). Even within the SNpc dopaminergic population, susceptibility to LP formation is not equally distributed, but is much higher in the ventral tier than in dorsal and medial tiers (Dickson, 2012; Wakabayashi et al., 2007).

What cell-type specific factors might control release and uptake of misfolded aSYN from the extracellular space (Fares et al., 2021)? One factor contributing to uptake that has already been mentioned is neuronal activity. Regenerative spike activity promotes PFF internalization from the extracellular space through a form of micropinocytosis (Ueda et al., 2021; Wu et al., 2020). How activity regulates this process is unclear, although Ca^2+^ entry is likely to be a factor. Ca^2+^ dependent release of exosomes containing aSYN also has been reported (Emmanouilidou et al., 2010). That said, there are many very active neurons in the brain (e.g., SNr neurons, cerebellar Purkinje neurons) that never manifest LP; whether they take up PFFs remains to be determined. Another factor contributing to uptake are putative surface receptors like LAG3, neurexin 1b, Aβ precursor-like protein 1, prion protein, or the α3-subunit of the Na^+^/K^+^-ATPase (Aulic et al., 2017; Mao et al., 2016; Shrivastava et al., 2015) and heparan sulfate proteoglycan mediated micropinocytosis (Holmes et al., 2013). However, it is unclear to what extent neuronal internalization can be attributed to receptor mediated endocytosis; for example, other groups have found that LAG3 is not expressed in neurons and therefore does not modulate propagation of aSYN pathology between neurons (Emmenegger et al., 2021). Transfer of aSYN pathology through tunnelling nanotubes, which may be created between specific cell types, also has been suggested (Abounit et al., 2016), and was recently shown to mediate transfer of aSYN fibrils between microglia (Scheiblich et al., 2021).

What other cell type-specific factors might contribute to the growth and persistence of intracellular aggregates? For misfolded aSYN that is taken up into the endosomal system, transit to the cytosol (where LP is found) is not well understood (Fares et al., 2021), despite evidence that endosomes/lysosomes may rupture (Jiang et al., 2017). Once released into the cytosol, what drives aggregation? At least in part, an answer might come from a consideration of what promotes fibril formation in the test tube. Two factors that have relevance are the concentration of aSYN, and divalent ions (like Ca^2+^) that charge shield the carboxyl terminal region of aSYN (Nath et al., 2011; Rcom-H'cheo-Gauthier et al., 2016). As noted above, the expression level of aSYN is correlated with PFF-induced spreading of pathology in rodent brain (Henderson et al., 2019a), primary neurons (Courte et al., 2020), and in PD (Erskine et al., 2018). Indeed, a key trait of the neurons that are most vulnerable to death in PD – dopaminergic SNpc neurons, noradrenergic LC neurons, serotonergic raphe neurons, cholinergic PPN and cholinergic basal forebrain neurons is that they have a massive axonal arbor with long and highly branched axons invested with transmitter release sites. In the human SNpc, the number of release sites is estimated to 1–2 million for one individual axon (Bolam and Pissadaki, 2012; Diederich et al., 2019). With this massive axon must come elevated expression of the presynaptic protein aSYN. Consistent with this idea, decreasing the axonal size of the SNpc dopaminergic neurons in culture significantly reduces their vulnerability to PD-linked stressors (Pacelli et al., 2015). Relatively low aSYN expression levels in regions like the cerebellum, SNr, and GPe could very well be a major factor in their resistance to LP (Courte et al., 2020; Taguchi et al., 2019).

In addition to having elevated expression of aSYN, many of the most vulnerable neurons in PD have high basal levels of cytosolic Ca^2+^, a divalent cation which may promote aggregation (Rcom-H'cheo-Gauthier et al., 2016). At-risk neurons in the SNpc, LC, basal forebrain, PPN, and DMV are slow autonomous pacemakers with broad action potentials (which promote Ca^2+^ entry), low intrinsic Ca^2+^ buffering and large activity-dependent fluctuations in cytosolic Ca^2+^ concentration (Dryanovski et al., 2013; Goldberg et al., 2012; Guzman et al., 2010; Sanchez-Padilla et al., 2014). In SNpc dopaminergic neurons and LC noradrenergic neurons, where this has been studied in more depth, the cytosolic Ca^2+^ oscillation is triggered by plasma membrane voltage-dependent Ca^2+^ channels with a Ca_v_1.3 pore-forming subunit. As in other excitable cells, these L-type channels are juxtaposed to ryanodine receptors (RYRs) in the endoplasmic reticulum (ER) and induce Ca^2+^-induced Ca^2+^ release (CICR) (Chan et al., 2007; Diaz-Vegas et al., 2018; Guzman et al., 2009; Viola et al., 2009). CICR promotes Ca^2+^ loading of mitochondria at specialized junctions (mitochondria-associated membranes). This loading stimulates oxidative phosphorylation (ATP production) by activating the malate-aspartate shuttle and disinhibiting matrix tricarboxylic acid cycle enzymes. As in muscle (Diaz-Vegas et al., 2018; Viola et al., 2009), this pathway serves as a feed-forward control mechanism for mitochondrial ATP production that anticipates demand, rather than waiting for ATP levels to fall and disinhibit mitochondrial complex V (Zampese and Surmeier, 2020). This should help to ensure that in times of stress or continuous demand, these critical neurons, which modulate large swaths of motor and cognitive circuitry do not shut down (Surmeier et al., 2017).

This feed-forward control comes at a cost. The electron flux necessary for oxidative phosphorylation generates reactive oxygen species, particularly at times when the stimulation precedes demand and mitochondrial membrane potential is high (Brookes et al., 2004). Indeed, basal mitochondrial oxidant stress in SNpc, LC, DMV and PPN neurons is high, particularly in dendrites and axons (Goldberg et al., 2012; Guzman et al., 2010; Sanchez-Padilla et al., 2014). Reactive oxygen and nitrogen species generation are associated with mitochondrial DNA deletions (Bender et al., 2006; Dolle et al., 2016; Sanders et al., 2014) and the loss of mitochondrial complex I function (Reeve et al., 2014; Schapira et al., 1990). This stress may be exacerbated by iron accumulation and the Fenton reaction in some at-risk neurons (Berg and Hochstrasser, 2006). Indeed, basal mitochondrial stress in SNpc dopaminergic neurons is sufficient to increase turnover of mitochondrial proteins (Guzman et al., 2018). In addition to damaging mitochondrial proteins, mitochondrial oxidant stress compromises lysosomal function (Burbulla et al., 2017) and may contribute to disruption of chaperone-mediated autophagy (Martinez-Vicente and Cuervo, 2007), both of which are linked to degradation of aSYN. Moreover, the Ca^2+^ signaling in vulnerable neurons leads to pacemaking-associated oscillations in dendritic Ca^2+^ concentration that may reach the micromolar level necessary to activate the calcineurin and protease calpain, both of which promote aggregation (Dehay et al., 2010; Diepenbroek et al., 2014; Dufty et al., 2007).

Another factor that may contribute to the vulnerability of dopaminergic neurons is the reliance upon dopamine (DA) as a transmitter. The combination of DA, Ca^2+^ and aSYN promotes the degeneration of murine dopaminergic neurons in vitro (Mosharov et al., 2009). DA modification of aSYN also blocks chaperone mediated autophagy (Martinez-Vicente et al., 2008). In human iPSC-derived dopaminergic neurons, mitochondrial oxidant stress drives oxidation of DA, which promotes lysosomal dysfunction and aSYN aggregation (Burbulla et al., 2017). Interestingly, in human neurons, cytosolic DA concentrations were higher than those in rodent-derived neurons, creating a condition that could underlie increased mitochondrial oxidant stress and neuromelanin accumulation (Burbulla et al., 2017; Graves et al., 2020). Although DA metabolism to neuromelanin has long been thought to protect dopaminergic neurons by sequestering reactive DA metabolites (Zucca et al., 2017), recent work has shown that too much neuromelanin can be toxic, probably as a consequence of crowding-induced inhibition of proteostasis (Carballo-Carbajal et al., 2019).

Thus, there may be a ‘perfect storm’ in vulnerable neurons with aging: 1) high levels of aSYN, Ca^2+^, reactive oxygen species (and DA in SNc neurons) which create a cellular environment that promotes aSYN aggregation and 2) flagging mechanisms for aggregate clearance. This possibility is supported by what we know about familial forms of PD (Kalia et al., 2015; Pickrell and Youle, 2015; Poulopoulos et al., 2012). Moreover, as mentioned above, partial deletion of Engrailed 1 in mice leads to exacerbated aSYN pathology (Chatterjee et al., 2019).

A related question is whether LP is an essential precondition for the development of PD symptomatology, or parkinsonism in broader terms? Duplications or triplications of the *SNCA* gene (PARK4) as well as point mutations (PARK1), like A53T, A30P, or E46K, are generally associated with early onset cPD and widespread LP (Poulopoulos et al., 2012). However, in PD cases driven by loss-of-function mutations in PINK1, PARK2, or PARK7, LP is sparse or absent (Doherty et al., 2013; Poulopoulos et al., 2012). Patients carrying a mutation in LRRK2 (encoding leucine-rich repeat serine/threonine-protein kinase 2), the most common autosomal-dominant cause of PD, have fairly heterogenous neuropathology, and LP is absent in a significant number of them (Hasegawa et al., 2009; Henderson et al., 2019c; Kalia et al., 2015; Poulopoulos et al., 2012; Ross et al., 2006; Wszolek et al., 2004). Dopaminergic neurons are also susceptible to age-related loss in the absence of disease (Collier et al., 2011; Giguere et al., 2018). These findings show that LP is not necessary for the development of parkinsonism or motor abnormalities.

While in this review we focus on neuronal factors that might play a role in dictating the propagation of LB pathology between brain regions, emerging findings suggest that glial cells also exhibit differences between brain regions (Boisvert et al., 2018; Clarke et al., 2018; Grabert et al., 2016; Soreq et al., 2017). Because both microglia and astrocytes have been suggested to influence both toxicity and cell-to-cell transfer of pathogenic aSYN conformers (Liddelow et al., 2017; Scheiblich et al., 2021; Yun et al., 2018), they too might influence how LP spreads between brain structures.

## 5. A mixed model of the spreading of aSYN pathology and future directions

Taken together, there is unequivocal evidence that fibrillar forms of aSYN can – once injected in the brain, gut, or muscle – spread along the connectome and seed LP-like pathology in mice, rats, and non-human primates. But the pattern of propagated pathology within these models does not simply reflect the synaptic connectome of neurons at the seeding site (Henderson et al., 2019a; Henrich et al., 2020). Rather, the distribution (and persistence) of aSYN pathology appears to be modulated by cell-autonomous and regional factors.

**Fig. 1.**
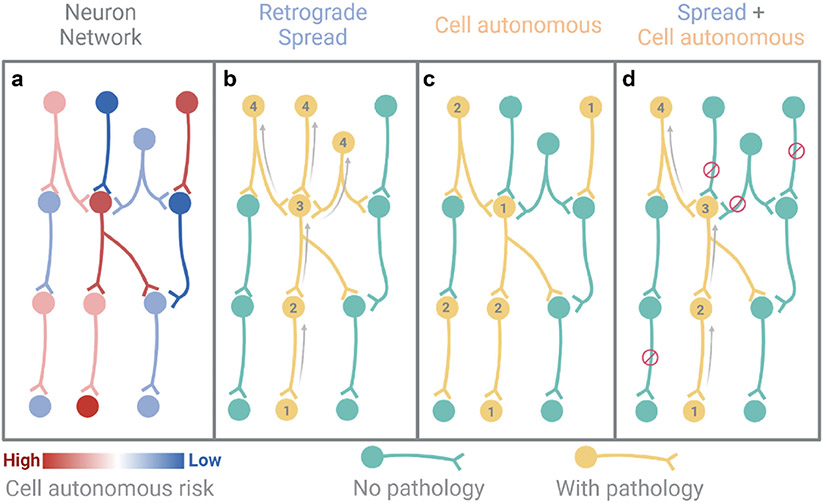
A mixed model of the spreading of aSYN pathology (a) We consider a very basic neuron network in which each neuron has an intrinsic risk to develop pathology based on cell autonomous factors discussed in the review. (b) Spread through connectivity (in this case in a retrograde direction) is useful to describe the pattern of pathology progression, such that if pathology started at neuron 1, we can estimate which neurons should subsequently exhibit pathology (1>2>3>4). However, if pathology were to spread along all anatomical connections, it would affect all connected regions, including many regions that never exhibit pathology. (c) Cell autonomous factors are useful in describing why some neurons are vulnerable within the network. However, if cell autonomous factors alone drove disease, it would seem likely that all vulnerable neurons would get pathology around the same time (1,1,1,2,2,2) in accordance with their cell autonomous risk. (d) Current evidence points to a mixed model where connectivity provides a conduit for pathology progression through the brain, but cell autonomous factors modulate which regions along that conduit exhibit pathology and which do not. This model posits that neurons that do not express cell autonomous vulnerability factors or are not connected to neurons with pathology will not develop pathology or will do so at a much slower rate. Graphic created with BioRender.com.

If we consider a simple model of aSYN spreading (Fig. 1a), there are several inferences that can be drawn. First, neuronal connectivity constrains and helps predict the rate at which aSYN pathology spreads. That is, the further away you are in the network, the longer it will take to possibly reach you (Fig. 1b). If connectivity was the only driver of pathology spread, pathology would be expected to reach nearly every cell in the brain eventually. This does not happen. Hence, cell autonomous or regional factors must impact either the development of aSYN pathology or its persistence (Fig 1c).

However, it is difficult to imagine how cell-autonomous factors alone could produce a pattern of pathology consistent with that observed in PD. First, neurons with certain vulnerability factors (high levels of aSYN, basal oxidant stress and elevated cytosolic Ca^2+^ levels, limited ‘spare’ capacity to degrade misfolding proteins because of the proteostatic demands placed on them by their axon and high rate of mitochondrial turnover) would all be predicted to manifest pathology. Indeed, with normal healthy aging, SNpc dopaminergic neurons are progressively lost (Giguere et al., 2018). That said, most people do not develop PD in their lifetimes, suggesting that other factors must accelerate the naturally occurring loss enough to reach the threshold for clinical manifestation. It is also important to note that some neurons that do not appear to have the traits of the most vulnerable neurons, do eventually manifest LP (e. g., cortical pyramidal neurons). While the impact of aging on these neurons may result in at least a subset of the factors linked to vulnerability (e.g., metabolic stress and elevated Ca^2+^ levels Rcom-H'cheo-Gauthier et al., 2016; Collier et al., 2011), a more parsimonious hypothesis is that LP in these neurons reflects spreading from another seeding site.

Thus, a ‘mixed’ model of LP that involves both propagation and cell autonomous factors has great appeal. This model posits that: 1) neuroanatomic connectivity constrains the spatiotemporal parameters of spreading, but 2) the persistence, magnitude and cellular consequences of the pathology are determined by local factors (Fig. 1d). This model does not exclude the possibility that aSYN pathology arises independently of propagated pathology, resulting in spread from those central sites, rather than the periphery. Recent work examining the longitudinal distribution of tau pathology in Alzheimer's disease also is consistent with a mixed model involving both trans-synaptic spreading and local factors (Meisl et al., 2021).

Although a great deal of progress has been made, there are several key questions that remain unanswered. One of these is to what extent PFF seeding reproduces the processes responsible for human LP (Fares et al., 2021). Once taken up, do PFFs produce human-like LP, which not only contains misfolded aSYN, but mitochondrial components, lipids and other molecules? Given that most spreading appears to be retrograde, do PFFs need to be modified by local cells (microglia, astrocytes, neurons) once deposited for there to be significant axon terminal uptake? Do cell-specific mechanisms limit terminal uptake? For example, does activity drive internalization at terminals the way it appears to at cell bodies? If there is retrograde propagation and somatodendritic release of misfolded aSYN, then what are the factors governing release?

It also remains to be determined whether the cell autonomous factors that promote aSYN pathology in humans actually affect PFF-induced pathology in model systems. There are a wide range of pharmacological, chemogenetic, optogenetic and molecular approaches that could be used to nail this question down. These same tools can be used to assess how aSYN pathology affects circuits implicated in motor and non-motor features of PD (Tozzi et al., 2021).

Another major question is whether the mechanisms mediating PFF induced neurodegeneration (which are poorly understood) mimic those found in human PD patients. PFF-induced neurodegeneration can be rapid in models, but neuronal loss appears to be very slow in humans (Huynh et al., 2021; McCann et al., 2016). Thus, it remains uncertain whether LP (or a linked aSYN pathology) actually causes neuronal death or is a disease ‘tombstone’ (Wakabayashi et al., 2007).

Getting clear answers to these questions (and others) should guide translational efforts to diminish LP and neuron death in humans suffering from PD. At present, most efforts to develop therapies that might diminish or reverse LP in humans are focused on enhancing lysosomal function, reducing inflammation or reducing the spread of misfolded aSYN with immunotherapies targeting aSYN (BIIB054, Prasinezumab; Phase II) (McFarthing et al., 2021). The latter therapy is based on the expectation that aSYN becomes extracellular and that clearing it may slow progression. The notion that blocking the uptake of pathogenic aSYN seeds with small molecules is possible has gained traction for reasons discussed above (Aulic et al., 2017; Emmenegger et al., 2021; Holmes et al., 2013; Mao et al., 2016; Shrivastava et al., 2015). That said, the extent to which these approaches will reverse existing pathology and restore function is unclear. Although several previous clinical trials have targeted cell autonomous mechanisms that might play a role in LP spreading, like Ca^2+^ entry through Cav1 channels, they have failed to yield significant results (McFarthing et al., 2021). A major issue in these trials has been whether there was adequate target engagement and biological efficacy of the therapeutic intervention. Recent re-examination of the STEADY-PD II and III trials suggest that there was a slowing of disease progression in those patients where there was more robust target engagement by isradipine (either because they were given an extended-release format of the drug or because they cleared it more slowly) (Surmeier et al., 2021; Venuto et al., 2021). Nevertheless, designing better strategies for manipulating cell autonomous factors that might contribute to pathogenesis in PD remains a challenge.

In summary, research in the last decade has dramatically improved our understanding of the potential mechanisms underlying the emergence and propagation of aSYN pathology in PD. The available data point to a mixed model in which both trans-synaptic propagation of aSYN pathology and cell autonomous mechanisms govern the pattern, magnitude and persistence of LP. Many questions remain unanswered regarding this mixed model. Filling these gaps in knowledge should facilitate the development of novel therapeutic strategies. We propose that strategies that target both propagation and cell autonomous determinants of aSYN pathology should prove more effective than targeting only one or the other.

## References

[R1] Aarsland D, Andersen K, Larsen JP, Lolk A, Kragh-Sorensen P, 2003. Prevalence and characteristics of dementia in Parkinson disease: an 8-year prospective study. Arch. Neurol 60, 387–392. 12633150 10.1001/archneur.60.3.387

[R2] Abdelmotilib H, Maltbie T, Delic V, Liu Z, Hu X, Fraser KB, Moehle MS, Stoyka L, Anabtawi N, Krendelchtchikova V, , 2017. Alpha-Synuclein fibril-induced inclusion spread in rats and mice correlates with dopaminergic neurodegeneration. Neurobiol. Dis 105, 84–98. 28576704 10.1016/j.nbd.2017.05.014 PMC5701756

[R3] Abounit S, Wu JW, Duff K, Victoria GS, Zurzolo C, 2016. Tunneling nanotubes: a possible highway in the spreading of tau and other prion-like proteins in neurodegenerative diseases. Prion 10, 344–351. 27715442 10.1080/19336896.2016.1223003 PMC5105909

[R4] Aulic S, Masperone L, Narkiewicz J, Isopi E, Bistaffa E, Ambrosetti E, Pastore B, De Cecco E, Scaini D, Zago P, , 2017. Alpha-Synuclein amyloids hijack prion protein to gain cell entry, facilitate cell-to-cell spreading and block prion replication. Sci. Rep 7, 10050. 28855681 10.1038/s41598-017-10236-x PMC5577263

[R5] Beach TG, Adler CH, Lue L, Sue LI, Bachalakuri J, Henry-Watson J, Sasse J, Boyer S, Shirohi S, Brooks R, , 2009. Unified staging system for Lewy body disorders: correlation with nigrostriatal degeneration, cognitive impairment and motor dysfunction. Acta Neuropathol. 117, 613–634. 19399512 10.1007/s00401-009-0538-8 PMC2757320

[R6] Bender A, Krishnan KJ, Morris CM, Taylor GA, Reeve AK, Perry RH, Jaros E, Hersheson JS, Betts J, Klopstock T, , 2006. High levels of mitochondrial DNA deletions in substantia nigra neurons in aging and Parkinson disease. Nat. Genet 38, 515–517. 16604074 10.1038/ng1769

[R7] Berg D, Hochstrasser H, 2006. Iron metabolism in parkinsonian syndromes. Movement Disorders 21, 1299–1310. 16817199 10.1002/mds.21020

[R8] Bezard E, Boraud T, Bioulac B, Gross CE, 1999. Involvement of the subthalamic nucleus in glutamatergic compensatory mechanisms. Eur. J. Neurosci 11, 2167–2170. 10336685 10.1046/j.1460-9568.1999.00627.x

[R9] Bezard E, Ravenscroft P, Gross CE, Crossman AR, Brotchie JM, 2001. Upregulation of striatal preproenkephalin gene expression occurs before the appearance of parkinsonian signs in 1-methyl-4-phenyl- 1,2,3,6-tetrahydropyridine monkeys. Neurobiol. Dis 8, 343–350. 11300729 10.1006/nbdi.2000.0375

[R10] Boisvert MM, Erikson GA, Shokhirev MN, Allen NJ, 2018. The aging astrocyte transcriptome from multiple regions of the mouse brain. Cell Rep. 22, 269–285. 29298427 10.1016/j.celrep.2017.12.039 PMC5783200

[R11] Bolam JP, Pissadaki EK, 2012. Living on the edge with too many mouths to feed: why dopamine neurons die. Movement Disorders 27, 1478–1483. 23008164 10.1002/mds.25135 PMC3504389

[R12] Borghammer P, 2021. The alpha-Synuclein origin and connectome model (SOC model) of Parkinson's disease: explaining motor asymmetry, non-motor phenotypes, and cognitive decline. J. Parkinsons Dis 11, 455–474. 33682732 10.3233/JPD-202481 PMC8150555

[R13] Braak H, Del Tredici K, 2009. Neuroanatomy and pathology of sporadic Parkinson's disease. Adv. Anat. Embryol. Cell Biol 201, 1–119. 19230552

[R14] Braak H, Del Tredici K, Rub U, de Vos RA, Jansen Steur EN, Braak E, 2003. Staging of brain pathology related to sporadic Parkinson's disease. Neurobiol. Aging 24, 197–211. 12498954 10.1016/s0197-4580(02)00065-9

[R15] Braak H, de Vos RA, Bohl J, Del Tredici K, 2006a. Gastric alpha-synuclein immunoreactive inclusions in Meissner’s and Auerbach’s plexuses in cases staged for Parkinson’s disease-related brain pathology. Neurosci. Lett 396, 67–72. 16330147 10.1016/j.neulet.2005.11.012

[R16] Braak H, Rub U, Del Tredici K, 2006b. Cognitive decline correlates with neuropathological stage in Parkinson’s disease. J. Neurol. Sci 248, 255–258. 16814807 10.1016/j.jns.2006.05.011

[R17] Brookes PS, Yoon Y, Robotham JL, Anders MW, Sheu SS, 2004. Calcium, ATP, and ROS: a mitochondrial love-hate triangle. Am J Physiol Cell Physiol 287, C817–C833. 15355853 10.1152/ajpcell.00139.2004

[R18] Brown JA, Deng J, Neuhaus J, Sible IJ, Sias AC, Lee SE, Kornak J, Marx GA, Karydas AM, Spina S, , 2019. Patient-tailored, connectivity-based forecasts of spreading brain atrophy. Neuron 104 (856–868), e855. 10.1016/j.neuron.2019.08.037 PMC7012373 31623919

[R19] Brundin P, Kordower JH, 2012. Neuropathology in transplants in Parkinson’s disease: implications for disease pathogenesis and the future of cell therapy. Prog. Brain Res 200, 221–241. 23195421 10.1016/B978-0-444-59575-1.00010-7

[R20] Brundin P, Melki R, 2017. Prying into the prion hypothesis for Parkinson’s disease. J. Neurosci 37, 9808–9818. 29021298 10.1523/JNEUROSCI.1788-16.2017 PMC5637113

[R21] Burbulla LF, Song P, Mazzulli JR, Zampese E, Wong YC, Jeon S, Santos DP, Blanz J, Obermaier CD, Strojny C, , 2017. Dopamine oxidation mediates mitochondrial and lysosomal dysfunction in Parkinson's disease. Science 357, 1255–1261. 28882997 10.1126/science.aam9080 PMC6021018

[R22] Carballo-Carbajal I, Laguna A, Romero-Gimenez J, Cuadros T, Bove J, Martinez-Vicente M, Parent A, Gonzalez-Sepulveda M, Penuelas N, Torra A, , 2019. Brain tyrosinase overexpression implicates age-dependent neuromelanin production in Parkinson’s disease pathogenesis. Nat. Commun 10, 973. 30846695 10.1038/s41467-019-08858-y PMC6405777

[R23] Chan CS, Guzman JN, Ilijic E, Mercer JN, Rick C, Tkatch T, Meredith GE, Surmeier DJ, 2007. Rejuvenation' protects neurons in mouse models of Parkinson's disease. Nature 447, 1081–1086. 17558391 10.1038/nature05865

[R24] Chatterjee D, Sanchez DS, Quansah E, Rey NL, George S, Becker K, Madaj Z, Steiner JA, Ma J, Escobar Galvis ML, , 2019. Loss of one Engrailed1 allele enhances induced alpha-Synucleinopathy. J. Parkinsons Dis 9, 315–326. 30932894 10.3233/JPD-191590 PMC6597991

[R25] Cheng HC, Ulane CM, Burke RE, 2010. Clinical progression in Parkinson disease and the neurobiology of axons. Ann. Neurol 67, 715–725. 20517933 10.1002/ana.21995 PMC2918373

[R26] Chu Y, Kordower JH, 2007. Age-associated increases of alpha-synuclein in monkeys and humans are associated with nigrostriatal dopamine depletion: is this the target for Parkinson’s disease? Neurobiol. Dis 25, 134–149. 17055279 10.1016/j.nbd.2006.08.021

[R27] Clarke LE, Liddelow SA, Chakraborty C, Munch AE, Heiman M, Barres BA, 2018. Normal aging induces A1-like astrocyte reactivity. Proc. Natl. Acad. Sci. U. S. A 115, E1896–E1905. 29437957 10.1073/pnas.1800165115 PMC5828643

[R28] Collier TJ, Kanaan NM, Kordower JH, 2011. Ageing as a primary risk factor for Parkinson’s disease: evidence from studies of non-human primates. Nat. Rev. Neurosci 12, 359–366. 21587290 10.1038/nrn3039 PMC3387674

[R29] Conway KA, Harper JD, Lansbury PT, 1998. Accelerated in vitro fibril formation by a mutant alpha-synuclein linked to early-onset Parkinson disease. Nat. Med 4, 1318–1320. 9809558 10.1038/3311

[R30] Courte J, Bousset L, Boxberg YV, Villard C, Melki R, Peyrin JM, 2020. The expression level of alpha-synuclein in different neuronal populations is the primary determinant of its prion-like seeding. Sci. Rep 10, 4895. 32184415 10.1038/s41598-020-61757-x PMC7078319

[R31] Dagher A, Zeighami Y, 2018. Testing the protein propagation hypothesis of Parkinson disease. Journal of experimental neuroscience 12 (1179069518786715). 30013389 10.1177/1179069518786715 PMC6043918

[R32] Dehay B, Bove J, Rodriguez-Muela N, Perier C, Recasens A, Boya P, Vila M, 2010. Pathogenic lysosomal depletion in Parkinson’s disease. J. Neurosci 30, 12535–12544. 20844148 10.1523/JNEUROSCI.1920-10.2010 PMC6633458

[R33] Diaz-Vegas AR, Cordova A, Valladares D, Llanos P, Hidalgo C, Gherardi G, De Stefani D, Mammucari C, Rizzuto R, Contreras-Ferrat A, , 2018. Mitochondrial calcium increase induced by RyR1 and IP3R channel activation after membrane depolarization regulates skeletal muscle metabolism. Front. Physiol 9, 791. 29988564 10.3389/fphys.2018.00791 PMC6026899

[R34] Dickson DW, 2012. Parkinson’s Disease and Parkinsonism: Neuropathology. Cold Spring Harbor Perspectives in Medicine 2. 10.1101/cshperspect.a009258 PMC3405828 22908195

[R35] Diederich NJ, James Surmeier D, Uchihara T, Grillner S, Goetz CG, 2019. Parkinson’s disease: is it a consequence of human brain evolution? Movement Disorders 34, 453–459. 30759321 10.1002/mds.27628 PMC6593760

[R36] Diepenbroek M, Casadei N, Esmer H, Saido TC, Takano J, Kahle PJ, Nixon RA, Rao MV, Melki R, Pieri L, , 2014. Overexpression of the calpain-specific inhibitor calpastatin reduces human alpha-Synuclein processing, aggregation and synaptic impairment in [A30P] alphaSyn transgenic mice. Hum. Mol. Genet 23, 3975–3989. 24619358 10.1093/hmg/ddu112 PMC4110482

[R37] Doherty KM, Silveira-Moriyama L, Parkkinen L, Healy DG, Farrell M, Mencacci NE, Ahmed Z, Brett FM, Hardy J, Quinn N, , 2013. Parkin disease: a clinicopathologic entity? JAMA neurology 70, 571–579. 23459986 10.1001/jamaneurol.2013.172 PMC4202385

[R38] Dolle C, Flones I, Nido GS, Miletic H, Osuagwu N, Kristoffersen S, Lilleng PK, Larsen JP, Tysnes OB, Haugarvoll K, , 2016. Defective mitochondrial DNA homeostasis in the substantia nigra in Parkinson disease. Nat. Commun 7, 13548. 27874000 10.1038/ncomms13548 PMC5121427

[R39] Dryanovski DI, Guzman JN, Xie Z, Galteri DJ, Volpicelli-Daley LA, Lee VM, Miller RJ, Schumacker PT, Surmeier DJ, 2013. Calcium entry and alpha-synuclein inclusions elevate dendritic mitochondrial oxidant stress in dopaminergic neurons. J. Neurosci 33, 10154–10164. 23761910 10.1523/JNEUROSCI.5311-12.2013 PMC3682382

[R40] Duffy PE, Tennyson VM, 1965. Phase and Electron microscopic observations of Lewy bodies and melanin granules in the substantia Nigra and locus Caeruleus in Parkinson’s disease*†. J. Neuropathol. Exp. Neurol 24, 398–414.

[R41] Dufty MF, Collier TJ, Patterson JR, Kemp CJ, Luk KC, Tansey MG, Paumier KL, Kanaan NM, Fischer DL, Polinski NK, , 2018. Lewy body-like alpha-synuclein inclusions trigger reactive microgliosis prior to nigral degeneration. J. Neuroinflammation 15, 129. 29716614 10.1186/s12974-018-1171-z PMC5930695

[R42] Dufty BM, Warner LR, Hou ST, Jiang SX, Gomez-Isla T, Leenhouts KM, Oxford JT, Feany MB, Masliah E, Rohn TT, 2007. Calpain-cleavage of alpha-synuclein: connecting proteolytic processing to disease-linked aggregation. Am. J. Pathol 170, 1725–1738. 17456777 10.2353/ajpath.2007.061232 PMC1854966

[R43] Dugger BN, Dickson DW, 2010. Cell type specific sequestration of choline acetyltransferase and tyrosine hydroxylase within Lewy bodies. Acta Neuropathol. 120, 633–639. 20721565 10.1007/s00401-010-0739-1 PMC3107979

[R44] Emmanouilidou E, Melachroinou K, Roumeliotis T, Garbis SD, Ntzouni M, Margaritis LH, Stefanis L, Vekrellis K, 2010. Cell-produced alpha-synuclein is secreted in a calcium-dependent manner by exosomes and impacts neuronal survival. J. Neurosci 30, 6838–6851. 20484626 10.1523/JNEUROSCI.5699-09.2010 PMC3842464

[R45] Emmenegger M, De Cecco E, Hruska-Plochan M, Eninger T, Schneider MM, Barth M, Tantardini E, de Rossi P, Bacioglu M, Langston RG, , 2021. LAG3 is not expressed in human and murine neurons and does not modulate alpha-synucleinopathies. EMBO Mol Med 13, e14745. 34309222 10.15252/emmm.202114745 PMC8422075

[R46] Erskine D, Patterson L, Alexandris A, Hanson PS, McKeith IG, Attems J, Morris CM, 2018. Regional levels of physiological alpha-synuclein are directly associated with Lewy body pathology. Acta Neuropathol. 135, 153–154. 29134319 10.1007/s00401-017-1787-6

[R47] Fares MB, Jagannath S, Lashuel HA, 2021. Reverse engineering Lewy bodies: how far have we come and how far can we go? Nat. Rev. Neurosci 22, 111–131. 33432241 10.1038/s41583-020-00416-6

[R48] Fearnley JM, Lees AJ, 1991. Ageing and Parkinson's disease: substantia nigra regional selectivity. Brain 114 (Pt 5), 2283–2301. 1933245 10.1093/brain/114.5.2283

[R49] Fereshtehnejad SM, Yao C, Pelletier A, Montplaisir JY, Gagnon JF, Postuma RB, 2019. Evolution of prodromal Parkinson's disease and dementia with Lewy bodies: a prospective study. Brain : a journal of neurology 142, 2051–2067. 31111143 10.1093/brain/awz111

[R50] Forno LS, 1996. Neuropathology of Parkinson’s disease. J. Neuropathol. Exp. Neurol 55, 259–272. 8786384 10.1097/00005072-199603000-00001

[R51] Giasson BI, Uryu K, Trojanowski JQ, Lee VM, 1999. Mutant and wild type human alpha-synucleins assemble into elongated filaments with distinct morphologies in vitro. J. Biol. Chem 274, 7619–7622. 10075647 10.1074/jbc.274.12.7619

[R52] Giguere N, Burke Nanni S, Trudeau LE, 2018. On cell loss and selective vulnerability of neuronal populations in Parkinson’s disease. Front. Neurol 9, 455. 29971039 10.3389/fneur.2018.00455 PMC6018545

[R53] Goedert M, Spillantini MG, Del Tredici K, Braak H, 2013. 100 years of Lewy pathology. Nat. Rev. Neurol 9, 13–24. 23183883 10.1038/nrneurol.2012.242

[R54] Goldberg JA, Guzman JN, Estep CM, Ilijic E, Kondapalli J, Sanchez-Padilla J, Surmeier DJ, 2012. Calcium entry induces mitochondrial oxidant stress in vagal neurons at risk in Parkinson’s disease. Nat. Neurosci 15, 1414–1421. 22941107 10.1038/nn.3209 PMC3461271

[R55] Grabert K, Michoel T, Karavolos MH, Clohisey S, Baillie JK, Stevens MP, Freeman TC, Summers KM, McColl BW, 2016. Microglial brain region-dependent diversity and selective regional sensitivities to aging. Nat. Neurosci 19, 504–516. 26780511 10.1038/nn.4222 PMC4768346

[R56] Graves SM, Xie Z, Stout KA, Zampese E, Burbulla LF, Shih JC, Kondapalli J, Patriarchi T, Tian L, Brichta L, , 2020. Dopamine metabolism by a monoamine oxidase mitochondrial shuttle activates the electron transport chain. Nat. Neurosci 23, 15–20. 31844313 10.1038/s41593-019-0556-3 PMC7257994

[R57] Greffard S, Verny M, Bonnet AM, Beinis JY, Gallinari C, Meaume S, Piette F, Hauw JJ, Duyckaerts C, 2006. Motor score of the unified Parkinson disease rating scale as a good predictor of Lewy body-associated neuronal loss in the substantia nigra. Arch. Neurol 63, 584–588. 16606773 10.1001/archneur.63.4.584

[R58] Greffard S, Verny M, Bonnet AM, Seilhean D, Hauw JJ, Duyckaerts C, 2010. A stable proportion of Lewy body bearing neurons in the substantia nigra suggests a model in which the Lewy body causes neuronal death. Neurobiol. Aging 31, 99–103. 18457903 10.1016/j.neurobiolaging.2008.03.015

[R59] Guzman JN, Sanchez-Padilla J, Chan CS, Surmeier DJ, 2009. Robust pacemaking in substantia nigra dopaminergic neurons. J. Neurosci 29, 11011–11019. 19726659 10.1523/JNEUROSCI.2519-09.2009 PMC2784968

[R60] Guzman JN, Sanchez-Padilla J, Wokosin D, Kondapalli J, Ilijic E, Schumacker PT, Surmeier DJ, 2010. Oxidant stress evoked by pacemaking in dopaminergic neurons is attenuated by DJ-1. Nature 468, 696–700. 21068725 10.1038/nature09536 PMC4465557

[R61] Guzman JN, Ilijic E, Yang B, Sanchez-Padilla J, Wokosin D, Galtieri D, Kondapalli J, Schumacker PT, Surmeier DJ, 2018. Systemic isradipine treatment diminishes calcium-dependent mitochondrial oxidant stress. J. Clin. Invest 128, 2266–2280. 29708514 10.1172/JCI95898 PMC5983329

[R62] Halliday GM, Song YJ, Harding AJ, 2011. Striatal beta-amyloid in dementia with Lewy bodies but not Parkinson’s disease. J. Neural Transm. (Vienna) 118, 713–719. 21479514 10.1007/s00702-011-0641-6

[R63] Hasegawa K, Stoessl AJ, Yokoyama T, Kowa H, Wszolek ZK, Yagishita S, 2009. Familial parkinsonism: study of original Sagamihara PARK8 (I2020T) kindred with variable clinicopathologic outcomes. Parkinsonism Relat. Disord 15, 300–306. 18804399 10.1016/j.parkreldis.2008.07.010 PMC2702757

[R64] Henderson MX, Chung CH, Riddle DM, Zhang B, Gathagan RJ, Seeholzer SH, Trojanowski JQ, Lee VMY, 2017. Unbiased proteomics of early Lewy body formation model implicates active microtubule affinity-regulating kinases (MARKs) in synucleinopathies. J. Neurosci 37, 5870–5884. 28522732 10.1523/JNEUROSCI.2705-16.2017 PMC5473205

[R65] Henderson MX, Cornblath EJ, Darwich A, Zhang B, Brown H, Gathagan RJ, Sandler RM, Bassett DS, Trojanowski JQ, Lee VMY, 2019a. Spread of alpha-synuclein pathology through the brain connectome is modulated by selective vulnerability and predicted by network analysis. Nat. Neurosci 22, 1248–1257. 31346295 10.1038/s41593-019-0457-5 PMC6662627

[R66] Henderson MX, Cornblath EJ, Darwich A, Zhang B, Brown H, Gathagan RJ, Sandler RM, Bassett DS, Trojanowski JQ, Lee VMY, 2019b. Spread of α-synuclein pathology through the brain connectome is modulated by selective vulnerability and predicted by network analysis. Nat. Neurosci 22, 1248–1257. 31346295 10.1038/s41593-019-0457-5 PMC6662627

[R67] Henderson MX, Sengupta M, Trojanowski JQ, Lee VMY, 2019c. Alzheimer's disease tau is a prominent pathology in LRRK2 Parkinson's disease. Acta neuropathologica communications 7, 183. 31733655 10.1186/s40478-019-0836-x PMC6858668

[R68] Henderson MX, Sedor S, McGeary I, Cornblath EJ, Peng C, Riddle DM, Li HL, Zhang B, Brown HJ, Olufemi MF, , 2020. Glucocerebrosidase activity modulates neuronal susceptibility to pathological alpha-Synuclein insult. Neuron 105 (822–836), e827. 10.1016/j.neuron.2019.12.004 PMC7060125 31899072

[R69] Henrich MT, Geibl FF, Lakshminarasimhan H, Stegmann A, Giasson BI, Mao X, Dawson VL, Dawson TM, Oertel WH, Surmeier DJ, 2020. Determinants of seeding and spreading of alpha-synuclein pathology in the brain. Sci. Adv 6. 10.1126/sciadv.abc2487 PMC7673735 33177086

[R70] Holmes BB, DeVos SL, Kfoury N, Li M, Jacks R, Yanamandra K, Ouidja MO, Brodsky FM, Marasa J, Bagchi DP, , 2013. Heparan sulfate proteoglycans mediate internalization and propagation of specific proteopathic seeds. Proc. Natl. Acad. Sci. U. S. A 110, E3138–E3147. 23898162 10.1073/pnas.1301440110 PMC3746848

[R71] Huynh B, Fu Y, Kirik D, Shine JM, Halliday GM, 2021. Comparison of locus Coeruleus pathology with Nigral and forebrain pathology in Parkinson’s disease. Mov. Disord 36, 2085–2093. 33899954 10.1002/mds.28615

[R72] Irwin DJ, White MT, Toledo JB, Xie SX, Robinson JL, Van Deerlin V, Lee VM, Leverenz JB, Montine TJ, Duda JE, , 2012. Neuropathologic substrates of Parkinson disease dementia. Ann. Neurol 72, 587–598. 23037886 10.1002/ana.23659 PMC3484250

[R73] Irwin DJ, Grossman M, Weintraub D, Hurtig HI, Duda JE, Xie SX, Lee EB, Van Deerlin VM, Lopez OL, Kofler JK, , 2017. Neuropathological and genetic correlates of survival and dementia onset in synucleinopathies: a retrospective analysis. The Lancet Neurology 16, 55–65. 27979356 10.1016/S1474-4422(16)30291-5 PMC5181646

[R74] Jiang P, Gan M, Yen SH, McLean PJ, Dickson DW, 2017. Impaired endolysosomal membrane integrity accelerates the seeding progression of alpha-synuclein aggregates. Sci. Rep 7, 7690. 28794446 10.1038/s41598-017-08149-w PMC5550496

[R75] Kalia LV, Lang AE, Hazrati LN, Fujioka S, Wszolek ZK, Dickson DW, Ross OA, Van Deerlin VM, Trojanowski JQ, Hurtig HI, , 2015. Clinical correlations with Lewy body pathology in LRRK2-related Parkinson disease. JAMA neurology 72, 100–105. 25401511 10.1001/jamaneurol.2014.2704 PMC4399368

[R76] Karpowicz RJ Jr., Haney CM, Mihaila TS, Sandler RM, Petersson EJ, Lee VM, 2017. Selective imaging of internalized proteopathic alpha-synuclein seeds in primary neurons reveals mechanistic insight into transmission of synucleinopathies. J. Biol. Chem 292, 13482–13497. 28611062 10.1074/jbc.M117.780296 PMC5555207

[R77] Kordower JH, Freeman TB, Snow BJ, Vingerhoets FJ, Mufson EJ, Sanberg PR, Hauser RA, Smith DA, Nauert GM, Perl DP, , 1995. Neuropathological evidence of graft survival and striatal reinnervation after the transplantation of fetal mesencephalic tissue in a patient with Parkinson’s disease. N. Engl. J. Med 332, 1118–1124. 7700284 10.1056/NEJM199504273321702

[R78] Kordower JH, Rosenstein JM, Collier TJ, Burke MA, Chen EY, Li JM, Martel L, Levey AE, Mufson EJ, Freeman TB, , 1996. Functional fetal nigral grafts in a patient with Parkinson’s disease: chemoanatomic, ultrastructural, and metabolic studies. J. Comp. Neurol 370, 203–230. 8808731 10.1002/(SICI)1096-9861(19960624)370:2<203::AID-CNE6>3.0.CO;2-6

[R79] Kordower JH, Chu Y, Hauser RA, Freeman TB, Olanow CW, 2008a. Lewy body-like pathology in long-term embryonic nigral transplants in Parkinson’s disease. Nat. Med 14, 504–506. 18391962 10.1038/nm1747

[R80] Kordower JH, Chu Y, Hauser RA, Olanow CW, Freeman TB, 2008b. Transplanted dopaminergic neurons develop PD pathologic changes: a second case report. Movement disorders : official journal of the Movement Disorder Society 23, 2303–2306. 19006193 10.1002/mds.22369

[R81] Kordower JH, Olanow CW, Dodiya HB, Chu Y, Beach TG, Adler CH, Halliday GM, Bartus RT, 2013. Disease duration and the integrity of the nigrostriatal system in Parkinson's disease. Brain : a journal of neurology 136, 2419–2431. 23884810 10.1093/brain/awt192 PMC3722357

[R82] Kurowska Z, Englund E, Widner H, Lindvall O, Li JY, Brundin P, 2011. Signs of degeneration in 12–22-year old grafts of mesencephalic dopamine neurons in patients with Parkinson’s disease. J. Parkinsons Dis 1, 83–92. 23939259 10.3233/JPD-2011-11004

[R83] Laansma MA, Bright JK, Al-Bachari S, Anderson TJ, Ard T, Assogna F, Baquero KA, Berendse HW, Blair J, Cendes F, , 2021. International multicenter analysis of brain structure across clinical stages of Parkinson’s disease. Movement Disorders 36, 2538–2594. 10.1002/mds.28706 PMC8595579 34288137

[R84] Lashuel HA, 2020. Do Lewy bodies contain alpha-synuclein fibrils? And does it matter? A brief history and critical analysis of recent reports. Neurobiol. Dis 141, 104876. 32339655 10.1016/j.nbd.2020.104876

[R85] Lewy FH, 1912. Paralysis Agitans. I. Pathologische Anatomie Handbuch der Neurologie.

[R86] Li JY, Englund E, Holton JL, Soulet D, Hagell P, Lees AJ, Lashley T, Quinn NP, Rehncrona S, Bjorklund A, , 2008. Lewy bodies in grafted neurons in subjects with Parkinson’s disease suggest host-to-graft disease propagation. Nat. Med 14, 501–503. 18391963 10.1038/nm1746

[R87] Li W, Englund E, Widner H, Mattsson B, van Westen D, Latt J, Rehncrona S, Brundin P, Bjorklund A, Lindvall O, , 2016. Extensive graft-derived dopaminergic innervation is maintained 24 years after transplantation in the degenerating parkinsonian brain. Proc. Natl. Acad. Sci. U. S. A 113, 6544–6549. 27140603 10.1073/pnas.1605245113 PMC4988567

[R88] Liddelow SA, Guttenplan KA, Clarke LE, Bennett FC, Bohlen CJ, Schirmer L, Bennett ML, Munch AE, Chung WS, Peterson TC, , 2017. Neurotoxic reactive astrocytes are induced by activated microglia. Nature 541, 481–487. 28099414 10.1038/nature21029 PMC5404890

[R89] Luk KC, Song C, O'Brien P, Stieber A, Branch JR, Brunden KR, Trojanowski JQ, Lee VM, 2009. Exogenous alpha-synuclein fibrils seed the formation of Lewy body-like intracellular inclusions in cultured cells. Proc. Natl. Acad. Sci. U. S. A 106, 20051–20056. 19892735 10.1073/pnas.0908005106 PMC2785290

[R90] Luk KC, Kehm V, Carroll J, Zhang B, O'Brien P, Trojanowski JQ, Lee VM, 2012. Pathological alpha-synuclein transmission initiates Parkinson-like neurodegeneration in nontransgenic mice. Science 338, 949–953. 23161999 10.1126/science.1227157 PMC3552321

[R91] Luna E, Decker SC, Riddle DM, Caputo A, Zhang B, Cole T, Caswell C, Xie SX, Lee VMY, Luk KC, 2018. Differential alpha-synuclein expression contributes to selective vulnerability of hippocampal neuron subpopulations to fibril-induced toxicity. Acta Neuropathol. 135, 855–875. 29502200 10.1007/s00401-018-1829-8 PMC5955788

[R92] Mahul-Mellier AL, Burtscher J, Maharjan N, Weerens L, Croisier M, Kuttler F, Leleu M, Knott GW, Lashuel HA, 2020. The process of Lewy body formation, rather than simply alpha-synuclein fibrillization, is one of the major drivers of neurodegeneration. Proc. Natl. Acad. Sci. U. S. A 117, 4971–4982. 32075919 10.1073/pnas.1913904117 PMC7060668

[R93] Mao X, Ou MT, Karuppagounder SS, Kam TI, Yin X, Xiong Y, Ge P, Umanah GE, Brahmachari S, Shin JH, , 2016. Pathological alpha-synuclein transmission initiated by binding lymphocyte-activation gene 3. Science 353. 10.1126/science.aah3374 PMC5510615 27708076

[R94] Martinez-Vicente M, Cuervo AM, 2007. Autophagy and neurodegeneration: when the cleaning crew goes on strike. The Lancet Neurology 6, 352–361. 17362839 10.1016/S1474-4422(07)70076-5

[R95] Martinez-Vicente M, Talloczy Z, Kaushik S, Massey AC, Mazzulli J, Mosharov EV, Hodara R, Fredenburg R, Wu DC, Follenzi A, , 2008. Dopamine-modified alpha-synuclein blocks chaperone-mediated autophagy. J. Clin. Invest 118, 777–788. 18172548 10.1172/JCI32806 PMC2157565

[R96] Masuda-Suzukake M, Nonaka T, Hosokawa M, Oikawa T, Arai T, Akiyama H, Mann DM, Hasegawa M, 2013. Prion-like spreading of pathological alpha-synuclein in brain. Brain 136, 1128–1138. 23466394 10.1093/brain/awt037 PMC3613715

[R97] McCann H, Cartwright H, Halliday GM, 2016. Neuropathology of alpha-synuclein propagation and braak hypothesis. Mov. Disord 31, 152–160. 26340605 10.1002/mds.26421

[R98] McFarthing K, Rafaloff G, Baptista MAS, Wyse RK, Stott SRW, 2021. Parkinson’s disease drug therapies in the clinical trial pipeline: 2021 update. J. Parkinsons Dis 11, 891–903. 34151864 10.3233/JPD-219006 PMC8461678

[R99] Meisl G, Hidari E, Allinson K, Rittman T, DeVos SL, Sanchez JS, Xu CK, Duff KE, Johnson KA, Rowe JB, , 2021. In vivo rate-determining steps of tau seed accumulation in Alzheimer’s disease. Sci. Adv 7, eabh1448. 34714685 10.1126/sciadv.abh1448 PMC8555892

[R100] Mendez I, Sanchez-Pernaute R, Cooper O, Vinuela A, Ferrari D, Bjorklund L, Dagher A, Isacson O, 2005. Cell type analysis of functional fetal dopamine cell suspension transplants in the striatum and substantia nigra of patients with Parkinson's disease. Brain : a journal of neurology 128, 1498–1510. 15872020 10.1093/brain/awh510 PMC2610438

[R101] Mezias C, Rey N, Brundin P, Raj A, 2019. Neural connectivity predicts spreading of alpha-synuclein pathology in fibril-injected mouse models: involvement of retrograde and anterograde axonal propagation. Neurobiol. Dis 134, 104623. 31628991 10.1016/j.nbd.2019.104623 PMC7138530

[R102] Mosharov EV, Larsen KE, Kanter E, Phillips KA, Wilson K, Schmitz Y, Krantz DE, Kobayashi K, Edwards RH, Sulzer D, 2009. Interplay between cytosolic dopamine, calcium, and alpha-synuclein causes selective death of substantia nigra neurons. Neuron 62, 218–229. 19409267 10.1016/j.neuron.2009.01.033 PMC2677560

[R103] Nandhagopal R, Kuramoto L, Schulzer M, Mak E, Cragg J, McKenzie J, McCormick S, Ruth TJ, Sossi V, de la Fuente-Fernandez R, , 2011. Longitudinal evolution of compensatory changes in striatal dopamine processing in Parkinson's disease. Brain : a journal of neurology 134, 3290–3298. 22075521 10.1093/brain/awr233

[R104] Nath S, Goodwin J, Engelborghs Y, Pountney DL, 2011. Raised calcium promotes alpha-synuclein aggregate formation. Mol. Cell. Neurosci 46, 516–526. 21145971 10.1016/j.mcn.2010.12.004

[R105] Olanow CW, Goetz CG, Kordower JH, Stoessl AJ, Sossi V, Brin MF, Shannon KM, Nauert GM, Perl DP, Godbold J, , 2003. A double-blind controlled trial of bilateral fetal nigral transplantation in Parkinson’s disease. Ann. Neurol 54, 403–414. 12953276 10.1002/ana.10720

[R106] Pacelli C, Giguere N, Bourque MJ, Levesque M, Slack RS, Trudeau LE, 2015. Elevated mitochondrial bioenergetics and axonal Arborization size are key contributors to the vulnerability of dopamine neurons. Current biology: CB 25, 2349–2360. 26320949 10.1016/j.cub.2015.07.050

[R107] Pandya S, Zeighami Y, Freeze B, Dadar M, Collins DL, Dagher A, Raj A, 2019. Predictive model of spread of Parkinson's pathology using network diffusion. Neuroimage 192, 178–194. 30851444 10.1016/j.neuroimage.2019.03.001 PMC7180066

[R108] Parkinson J, 2002. An essay on the shaking palsy. 1817. The Journal of neuropsychiatry and clinical neurosciences 14, 223–236 (discussion 222). 11983801 10.1176/jnp.14.2.223

[R109] Peng C, Gathagan RJ, Covell DJ, Medellin C, Stieber A, Robinson JL, Zhang B, Pitkin RM, Olufemi MF, Luk KC, , 2018. Cellular milieu imparts distinct pathological alpha-synuclein strains in alpha-synucleinopathies. Nature. 557, 558–563. 29743672 10.1038/s41586-018-0104-4 PMC5970994

[R110] Pickrell AM, Youle RJ, 2015. The roles of PINK1, parkin, and mitochondrial fidelity in Parkinson's disease. Neuron 85, 257–273. 25611507 10.1016/j.neuron.2014.12.007 PMC4764997

[R111] Pienaar IS, Elson JL, Racca C, Nelson G, Turnbull DM, Morris CM, 2013. Mitochondrial abnormality associates with type-specific neuronal loss and cell morphology changes in the pedunculopontine nudeus in Parkinson disease. Am. J. Pathol 183, 1826–1840. 24099985 10.1016/j.ajpath.2013.09.002 PMC4188170

[R112] Postuma RB, Berg D, 2019. Prodromal Parkinson’s disease: the decade past, the decade to come. Movement disorders : official journal of the Movement Disorder Society 34, 665–675. 30919499 10.1002/mds.27670

[R113] Poulopoulos M, Levy OA, Alcalay RN, 2012. The neuropathology of genetic Parkinson's disease. Movement disorders : official journal of the Movement Disorder Society 27, 831–842. 22451330 10.1002/mds.24962 PMC3383342

[R114] Prusiner SB, Woerman AL, Mordes DA, Watts JC, Rampersaud R, Berry DB, Patel S, Oehler A, Lowe JK, Kravitz SN, , 2015. Evidence for alpha-synuclein prions causing multiple system atrophy in humans with parkinsonism. Proc. Natl. Acad. Sci. U. S. A 112, E5308–E5317. 26324905 10.1073/pnas.1514475112 PMC4586853

[R115] Rcom-H’cheo-Gauthier AN, Osborne SL, Meedeniya AC, Pountney DL, 2016. Calcium: alpha-Synuclein interactions in alpha-Synucleinopathies. Front. Neurosci 10, 570. 28066161 10.3389/fnins.2016.00570 PMC5167751

[R116] Recasens A, Dehay B, Bove J, Carballo-Carbajal I, Dovero S, Perez-Villalba A, Fernagut PO, Blesa J, Parent A, Perier C, , 2014. Lewy body extracts from Parkinson disease brains trigger alpha-synuclein pathology and neurodegeneration in mice and monkeys. Ann. Neurol 75, 351–362. 24243558 10.1002/ana.24066

[R117] Reeve A, Simcox E, Turnbull D, 2014. Ageing and Parkinson’s disease: why is advancing age the biggest risk factor? Ageing Res. Rev 14, 19–30. 24503004 10.1016/j.arr.2014.01.004 PMC3989046

[R118] Rey NL, George S, Brundin P, 2016a. Review: spreading the word: precise animal models and validated methods are vital when evaluating prion-like behaviour of alpha-synuclein. Neuropathol. Appl. Neurobiol 42, 51–76. 26666838 10.1111/nan.12299

[R119] Rey NL, Steiner JA, Maroof N, Luk KC, Madaj Z, Trojanowski JQ, Lee VM, Brundin P, 2016b. Widespread transneuronal propagation of alpha-synucleinopathy triggered in olfactory bulb mimics prodromal Parkinson’s disease. J. Exp. Med 213, 1759–1778. 27503075 10.1084/jem.20160368 PMC4995088

[R120] Rey NL, George S, Steiner JA, Madaj Z, Luk KC, Trojanowski JQ, Lee VM, Brundin P, 2017. Spread of aggregates after olfactory bulb injection of alpha-synuclein fibrils is associated with early neuronal loss and is reduced long term. Acta (neuropathologica) 135, 65–83. 29209768 10.1007/s00401-017-1792-9 PMC5756266

[R121] Rey NL, Bousset L, George S, Madaj Z, Meyerdirk L, Schulz E, Steiner JA, Melki R, Brundin P, 2019. Alpha-Synuclein conformational strains spread, seed and target neuronal cells differentially after injection into the olfactory bulb. Acta neuropathologica communications 7, 221. 31888771 10.1186/s40478-019-0859-3 PMC6937797

[R122] Ross OA, Toft M, Whittle AJ, Johnson JL, Papapetropoulos S, Mash DC, Litvan I, Gordon MF, Wszolek ZK, Farrer MJ, , 2006. Lrrk2 and Lewy body disease. Ann. Neurol 59, 388–393. 16437559 10.1002/ana.20731

[R123] Sanchez-Padilla J, Guzman JN, Ilijic E, Kondapalli J, Galtieri DJ, Yang B, Schieber S, Oertel W, Wokosin D, Schumacker PT, , 2014. Mitochondrial oxidant stress in locus coeruleus is regulated by activity and nitric oxide synthase. Nat. Neurosci 17, 832–840. 24816140 10.1038/nn.3717 PMC4131291

[R124] Sanders LH, McCoy J, Hu X, Mastroberardino PG, Dickinson BC, Chang CJ, Chu CT, Van Houten B, Greenamyre JT, 2014. Mitochondrial DNA damage: molecular marker of vulnerable nigral neurons in Parkinson’s disease. Neurobiol. Dis 70, 214–223. 24981012 10.1016/j.nbd.2014.06.014 PMC4144978

[R125] Schaeffer E, Postuma RB, Berg D, 2020. Prodromal PD: a new nosological entity. Prog. Brain Res 252, 331–356. 32247370 10.1016/bs.pbr.2020.01.003

[R126] Schapira AH, Cooper JM, Dexter D, Clark JB, Jenner P, Marsden CD, 1990. Mitochondrial complex I deficiency in Parkinson’s disease. J. Neurochem 54, 823–827. 2154550 10.1111/j.1471-4159.1990.tb02325.x

[R127] Scheiblich H, Dansokho C, Mercan D, Schmidt SV, Bousset L, Wischhof L, Eikens F, Odainic A, Spitzer J, Griep A, , 2021. Microglia jointly degrade fibrillar alpha-synuclein cargo by distribution through tunneling nanotubes. Cell 184 (5089–5106), e5021. 10.1016/j.cell.2021.09.007 PMC8527836 34555357

[R128] Schwarz LA, Miyamichi K, Gao XJ, Beier KT, Weissbourd B, DeLoach KE, Ren J, Ibanes S, Malenka RC, Kremer EJ, , 2015. Viral-genetic tracing of the input-output organization of a central noradrenaline circuit. Nature 524, 88–92. 26131933 10.1038/nature14600 PMC4587569

[R129] Schweighauser M, Shi Y, Tarutani A, Kametani F, Murzin AG, Ghetti B, Matsubara T, Tomita T, Ando T, Hasegawa K, , 2020. Structures of alpha-synuclein filaments from multiple system atrophy. Nature 585, 464–469. 32461689 10.1038/s41586-020-2317-6 PMC7116528

[R130] Shahmoradian SH, Lewis AJ, Genoud C, Hench J, Moors TE, Navarro PP, Castano-Diez D, Schweighauser G, Graff-Meyer A, Goldie KN, , 2019. Lewy pathology in Parkinson’s disease consists of crowded organelles and lipid membranes. Nat. Neurosci 22, 1099–1109. 31235907 10.1038/s41593-019-0423-2

[R131] Shrivastava AN, Redeker V, Fritz N, Pieri L, Almeida LG, Spolidoro M, Liebmann T, Bousset L, Renner M, Lena C, , 2015. Alpha-synuclein assemblies sequester neuronal alpha3-Na+/K+-ATPase and impair Na+ gradient. EMBO J. 34, 2408–2423. 26323479 10.15252/embj.201591397 PMC4601662

[R132] Soreq L, Consortium, U.K.B.E., North American Brain Expression, C, Rose J, Soreq E, Hardy J, Trabzuni D, Cookson MR, Smith C, Ryten M, , 2017. Major shifts in glial regional identity are a transcriptional Hallmark of human brain aging. Cell Rep. 18, 557–570. 28076797 10.1016/j.celrep.2016.12.011 PMC5263238

[R133] Spillantini MG, Schmidt ML, Lee VM, Trojanowski JQ, Jakes R, Goedert M, 1997. Alpha-synuclein in Lewy bodies. Nature 388, 839–840. 9278044 10.1038/42166

[R134] Spillantini MG, Crowther RA, Jakes R, Cairns NJ, Lantos PL, Goedert M, 1998a. Filamentous alpha-synuclein inclusions link multiple system atrophy with Parkinson’s disease and dementia with Lewy bodies. Neurosci. Lett 251, 205–208. 9726379 10.1016/s0304-3940(98)00504-7

[R135] Spillantini MG, Crowther RA, Jakes R, Hasegawa M, Goedert M, 1998b. Alpha-Synudein in filamentous inclusions of Lewy bodies from Parkinson's disease and dementia with lewy bodies. Proc. Natl. Acad. Sci. U. S. A 95, 6469–6473. 9600990 10.1073/pnas.95.11.6469 PMC27806

[R136] Surmeier DJ, Obeso JA, Halliday GM, 2017. Selective neuronal vulnerability in Parkinson disease. Nat. Rev. Neurosci 18, 101–113. 28104909 10.1038/nrn.2016.178 PMC5564322

[R137] Surmeier DJ, Nguyen JT, Lancki N, Venuto CS, Oakes D, Simuni TS, Wyse RK, 2021. Re-analysis of the STEADY-PD II trial—evidence for slowing the progression of Parkinson's disease. Mov. Disord 37, 334–342. 34766657 10.1002/mds.28850 PMC8922308

[R138] Taguchi K, Watanabe Y, Tsujimura A, Tanaka M, 2019. Expression of alpha-synuclein is regulated in a neuronal cell type-dependent manner. Anat. Sci. Int 94, 11–22. 30362073 10.1007/s12565-018-0464-8 PMC6315015

[R139] Tanudjojo B, Shaikh SS, Fenyi A, Bousset L, Agarwal D, Marsh J, Zois C, Heman-Ackah S, Fischer R, Sims D, , 2021. Phenotypic manifestation of alpha-synuclein strains derived from Parkinson’s disease and multiple system atrophy in human dopaminergic neurons. Nat. Commun 12, 3817. 34155194 10.1038/s41467-021-23682-z PMC8217249

[R140] Tozzi A, Sciaccaluga M, Loffredo V, Megaro A, Ledonne A, Cardinale A, Federici M, Bellingacci L, Paciotti S, Ferrari E, , 2021. Dopamine-dependent early synaptic and motor dysfunctions induced by alpha-synuclein in the nigrostriatal circuit. Brain 144, 3477–3491. 34297092 10.1093/brain/awab242 PMC8677552

[R141] Tran HT, Chung CH, Iba M, Zhang B, Trojanowski JQ, Luk KC, Lee VM, 2014. Alpha-synuclein immunotherapy blocks uptake and templated propagation of misfolded alpha-synuclein and neurodegeneration. Cell Rep. 7, 2054–2065. 24931606 10.1016/j.celrep.2014.05.033 PMC4410967

[R142] Ueda J, Uemura N, Sawamura M, Taguchi T, Ikuno M, Kaji S, Taruno Y, Matsuzawa S, Yamakado H, Takahashi R, 2021. Perampanel inhibits alpha-Synuclein transmission in Parkinson’s disease models. Movement Disorders 36, 1554–1564. 33813737 10.1002/mds.28558

[R143] Venuto CS, Yang L, Javidnia M, Oakes D, James Surmeier D, Simuni T, 2021. Isradipine plasma pharmacokinetics and exposure-response in early Parkinson's disease. Ann Clin Transl Neurol 8, 603–612. 33460320 10.1002/acn3.51300 PMC7951102

[R144] Viola HM, Arthur PG, Hool LC, 2009. Evidence for regulation of mitochondrial function by the L-type Ca2+ channel in ventricular myocytes. J. Mol. Cell. Cardiol 46, 1016–1026. 19166857 10.1016/j.yjmcc.2008.12.015

[R145] Volpicelli-Daley LA, Luk KC, Patel TP, Tanik SA, Riddle DM, Stieber A, Meaney DF, Trojanowski JQ, Lee VM, 2011. Exogenous alpha-synuclein fibrils induce Lewy body pathology leading to synaptic dysfunction and neuron death. Neuron 72, 57–71. 21982369 10.1016/j.neuron.2011.08.033 PMC3204802

[R146] Wakabayashi K, Tanji K, Mori F, Takahashi H, 2007. The Lewy body in Parkinson's disease: molecules implicated in the formation and degradation of alpha-synuclein aggregates. Neuropathology : official journal of the Japanese Society of Neuropathology 27, 494–506. 18018486 10.1111/j.1440-1789.2007.00803.x

[R147] Wszolek ZK, Pfeiffer RF, Tsuboi Y, Uitti RJ, McComb RD, Stoessl AJ, Strongosky AJ, Zimprich A, Muller-Myhsok B, Farrer MJ, , 2004. Autosomal dominant parkinsonism associated with variable synuclein and tau pathology. Neurology 62, 1619–1622. 15136696 10.1212/01.wnl.0000125015.06989.db

[R148] Wu Q, Shaikh MA, Meymand ES, Zhang B, Luk KC, Trojanowski JQ, Lee VM, 2020. Neuronal activity modulates alpha-synuclein aggregation and spreading in organotypic brain slice cultures and in vivo. Acta (neuropathologica) 140, 831–849. 33021680 10.1007/s00401-020-02227-6 PMC8030660

[R149] Yun SP, Kam TI, Panicker N, Kim S, Oh Y, Park JS, Kwon SH, Park YJ, Karuppagounder SS, Park H, , 2018. Block of A1 astrocyte conversion by microglia is neuroprotective in models of Parkinson’s disease. Nat. Med 24, 931–938. 29892066 10.1038/s41591-018-0051-5 PMC6039259

[R150] Zampese E, Surmeier DJ, 2020. Calcium, bioenergetics, and Parkinson’s disease. Cells 9. 10.3390/cells9092045 PMC7564460 32911641

[R151] Zheng T, Zhang Z, 2021. Activated microglia facilitate the transmission of alpha-synuclein in Parkinson’s disease. Neurochem. Int 148, 105094. 34097990 10.1016/j.neuint.2021.105094

[R152] Zucca FA, Segura-Aguilar J, Ferrari E, Munoz P, Paris I, Sulzer D, Sarna T, Casella L, Zecca L, 2017. Interactions of iron, dopamine and neuromelanin pathways in brain aging and Parkinson’s disease. Prog. Neurobiol 155, 96–119. 26455458 10.1016/j.pneurobio.2015.09.012 PMC4826627

